# Urban Parks Act as Refuges for Avian Biodiversity in Chaoyang District, Beijing (China)

**DOI:** 10.1002/ece3.73953

**Published:** 2026-07-02

**Authors:** Anees Ur Rahman, Luciano Bosso, Shahid Ahmad, Najeeb Ullah, Muhammad Zahid, Rashid Rasool Rabbani Ismaili

**Affiliations:** ^1^ School of Ecology and Nature Conservation Beijing Forestry University Beijing China; ^2^ Institute for Agriculture and Forestry Systems in the Mediterranean (ISAFoM) National Research Council of Italy Portici Italy; ^3^ Northeast Asia Biodiversity Research Center and Sino‐Russia Joint Center for Biodiversity Research Northeast Forestry University Harbin China; ^4^ College of Wildlife and Protected Area Northeast Forestry University Harbin China; ^5^ Department of Zoology Islamia College Peshawar Peshawar Pakistan; ^6^ Key Laboratory of Non‐Invasive Research Technology for Endangered Species Beijing Forestry University Beijing China

**Keywords:** bird communities, habitat heterogeneity, migration, NMDS ordination, special richness, urban biodiversity, wetland conservation

## Abstract

With the rapid expansion of urban areas, city parks play a vital role in sustaining biodiversity, particularly for birds. In this study, we examined bird species richness, seasonal dynamics, and habitat associations in four urban parks within Beijing's Chaoyang District (China): Olympic Forest Park, Chaoyang Park, Dongba Country Park, and Wenyu River Park. We carried out standardised point count surveys across 40 randomly selected plots. We recorded a total of 68 bird species, with Wenyu River Park supporting the highest diversity, likely due to its extensive wetlands and complex habitat structure. Seasonal changes in richness were most pronounced during the spring and fall migrations. We found that the wetland habitats were particularly important for several species, including the endangered *Anser cygnoides* and the threatened *Acrocephalus tangorum* . Non‐metric multidimensional scaling analysis revealed significant differences in bird community composition among the parks. Our findings underscore the vital role of urban greenspaces in maintaining both resident and migratory avian diversity. We recommend that urban planning prioritize the preservation and strategic management of wetlands and other heterogeneous habitats to safeguard biodiversity within urban landscapes.

## Graphical abstract

We assessed bird species richness, seasonal dynamics, and habitat associations across four urban parks in Beijing's Chaoyang District (China) using standardised point count surveys. A total of 68 bird species were recorded, with the highest diversity occurring in parks with extensive wetlands and marked seasonal variation during migration periods. Our results highlight the critical role of wetland and structurally complex habitats in sustaining resident and migratory bird communities within rapidly urbanising landscapes.

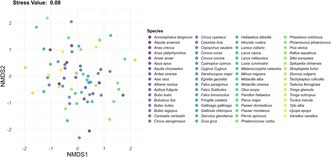

## 1 Introduction

Urban wildlife depends on green spaces for habitat and reproduction. In urban areas, birds play a crucial role, as they help control pest populations, pollinate plants and disperse seeds, thereby supporting the maintenance of green spaces (e.g., Yousefi et al. 2025). Laws can be used to protect bird populations in cities and to conserve biodiversity in areas that are becoming more developed (Cox et al. 2017). Various features, both near where birds live and throughout the landscape, are known to influence the richness of birds in cities (Aida et al. 2016; Beninde et al. 2015; Fraissinet et al. 2023). Differences in urban bird communities arise from the trade‐off between resource availability and environmental risks. Usually, suburban habitats support more bird species than dense urban cores, partly because of the greater diversity of available habitats, such as gardens, hedgerows, and edge vegetation, as well as the higher availability of invertebrate prey and the lower impervious surface cover (Lerman 2011; Beninde et al. 2015). Most research on urban birds is conducted in parks rather than in residential areas. Despite that, grounds and gardens around apartment complexes are also important for birds. Rapid urbanization in China poses threats to the preservation of biodiversity. However, research indicates that community‐managed green spaces can help mitigate environmental degradation (Lerman 2011). Strohbach et al. (2009) found that greater variation in urban bird communities can be explained by average green patch size, and even small green areas can build habitat connections among nearby larger spaces (e.g., Ahmad et al. 2025). Urban wetlands, which are often overlooked in urban planning, are crucial for many bird species that inhabit or migrate through urban areas (La Sorte et al. 2018). With the rapid growth of cities, wetlands are often lost or degraded, reducing their ability to support biodiversity (Goddard et al. 2010).

Urban green spaces should prioritize wetland preservation as the most effective way to ensure that wildlife and birds can move freely across the area (Aronson et al. 2017). Growing urbanization is being observed worldwide, and experts project that by 2050, up to 70% of the world's population will live in urban areas (UN 2012). Consequently, wildlife in urban areas experiences habitat fragmentation and faces a range of ecological pressures (Bergerot et al. 2011). There is considerable evidence that bird communities are altered by urban growth, often leading to a decrease in total species numbers and local extinctions (Chace and Walsh 2006; Ferenc et al. 2014).

The goal of our study was to assess the composition of bird communities in selected urban parks in the Chaoyang District of Beijing by employing Non‐metric MultiDimensional Scaling (NMDS) and PERMANOVA. In particular, the abundance and diversity of bird species in each park were evaluated, and we hypothesised that greater vegetation cover and the presence of water features would lead to higher bird species richness. We also investigated seasonal variation in bird communities and confirmed that migratory birds were most abundant during the spring and autumn migration seasons, as observed in other East Asian urban areas (La Sorte et al. 2018). Furthermore, we investigated how different habitat types within the parks—including areas combining trees and water with open spaces—influence bird distribution, anticipating that these heterogeneous habitats would host higher densities and greater species richness. Through these objectives, we aim to offer helpful information regarding the role of urban green spaces in promoting avian abundance, supporting biodiversity conservation and informing future urban park planning.

## 2 Materials and Methods

### 2.1 Study Area

**FIGURE 1:**
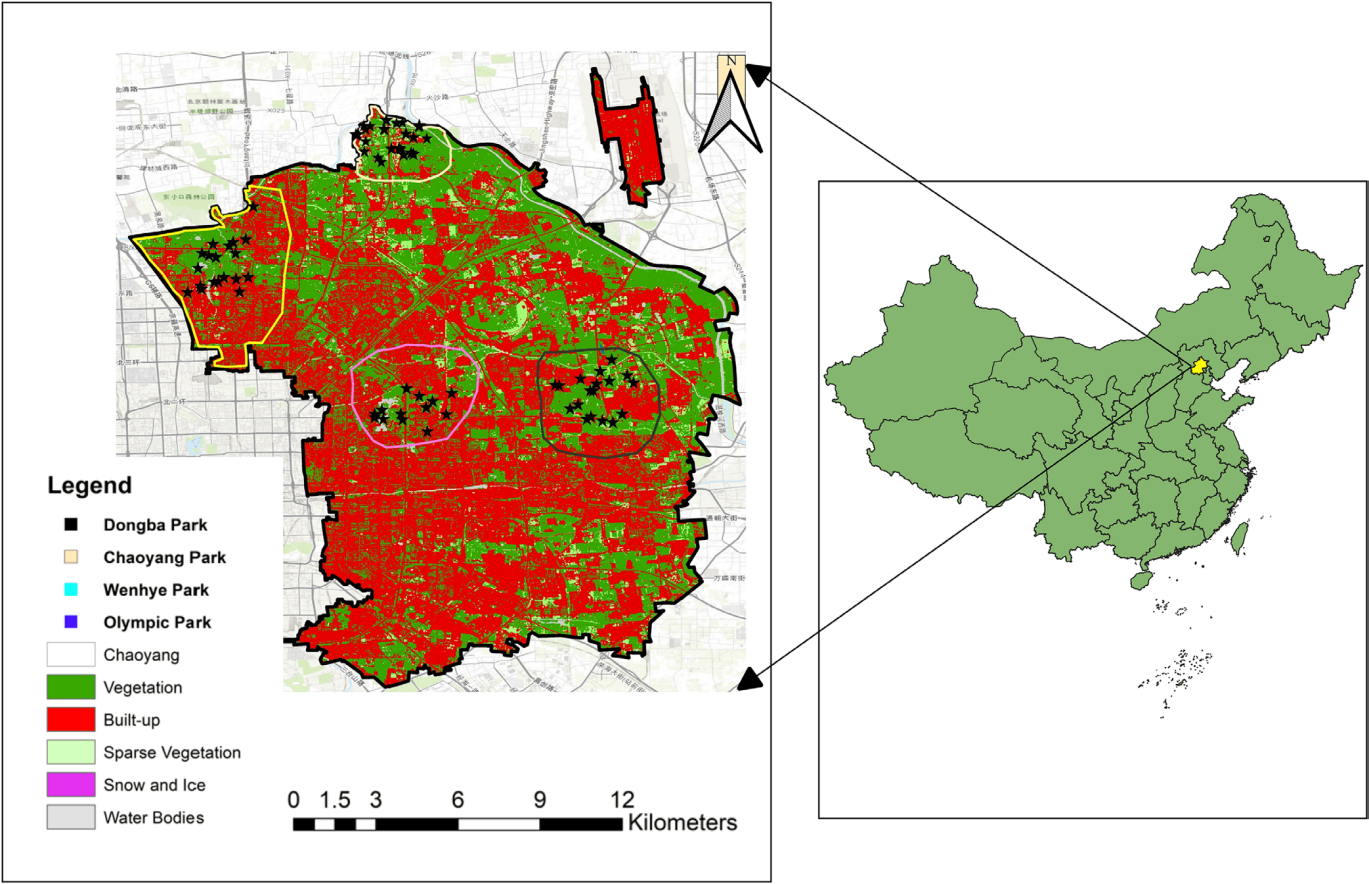
Map of the study area. The study area is shown with the four parks that were surveyed in the Chaoyang District of Beijing City (China, 39°54′ N, 116°28′ E). The approximate centroids of each of the study parks are indicated by black stars.

The distribution of habitats and the main parks in the Chaoyang District is shown in Figure 1. The study area is in the eastern part of Beijing (China, approximately 39°54′ N, 116°28′ E). The four study parks differ in age and origin: Olympic Forest Park was opened in 2008, Chaoyang Park in 1984, Dongba Country Park in the early 2000s, and Wenyu River Park was developed along the Wenyu River corridor, with its main habitats being restored and improved around 2007.

Seminatural features within the district are mainly associated with the Liangma and Tonghui rivers, which are partly canalised but still retain stretches of riparian vegetation that provide habitat for waterside bird species. According to land‐use statistics released by the Beijing Municipal Bureau of Statistics in 2022, approximately 70% of the district is urbanised.

The district covers approximately 478 km^2^ and reaches a maximum elevation of 60 m, characteristic of the flat terrain of the North China Plain. Chaoyang is highly urbanised, containing dense business districts, diplomatic residences, and numerous parks, while its natural features are largely represented by rivers such as the Liangma and Tonghui. Approximately 70% of the district is urbanised, with the remaining area occupied by green spaces, including public parks. Chaoyang is characterised by a humid continental climate, with summer temperatures exceeding 30°C and winter temperatures dropping below 0°C. This climate strongly influences local vegetation and bird abundance, shaping both the spatial distribution and seasonal movements of species within urban parks.

The study area comprises a mix of native and cultivated vegetation. *Populus* (poplars) dominate riparian zones, while *Ginkgo biloba* , a culturally significant ornamental tree, is widely planted for its resilience. resilience. The plant communities consist of native species (*Populus* and *Pinus tabuliformis* ) and introduced ornamentals and exotic turf grasses, with these species providing year‐round coniferous cover and soil protection and structural habitat complexity for cavity‐nesting birds (Qiang and Hu 2022).

### 2.2 Bird Surveys

Plots were established using a systematic random design: each park was divided into a 200 × 200 m grid, and one counting station was placed randomly within each grid cell. Where a randomly generated point fell in an inaccessible location (e.g., a building), it was replaced by the nearest accessible point within the same cell. We established 80 permanent sampling plots (20 per park), each centered on a fixed point count station. Stations were spaced 150–200 m apart with a 50 m fixed‐radius buffer. To reduce within‐session double counting, observers tracked everyone's position and excluded birds that flew into the plot from outside the buffer after the count began. Species identification relied on field guides, binoculars, and reference sound recordings. This protocol, aligned with and adapted from Rahman et al. (2024, 2025), allowed us to characterize and compare bird communities across the four parks. All four seasons of the year were sampled for species presence and abundance, and species richness (total number of species per plot) and species diversity (Shannon–Wiener index *H*′) were calculated with R using the vegan package (Oksanen et al. 2007).

The research was carried out in the field from April 2023 to March 2024 for 1 year. Surveys were done from 7 to 10 a.m., the time at which birds are most active. To observe species that are active in the afternoon, a supplementary afternoon session was held between 3:00 and 4:00 p.m. to capture more species, including waders and waterfowl that tend to be more active during late afternoon around Wenyu River Park's wetlands (Rahman et al. 2025). We recognize that afternoon point counts are atypical for point counting surveys and potentially underestimate some passerine species and state these findings as a limitation. To obtain a robust and representative picture of local environmental conditions, we repeated surveys four times during the study period, accounting for temporal variation in both bird and plant communities.

We identified bird species using both visual observations and auditory cues, supported by binoculars. To ensure accurate species identification, we compared observed vocalizations with reference sound recordings. This combined visual–acoustic approach, following the methodologies of Rahman et al. (2024, 2025), increased the reliability and accuracy of species detection.

Within the study area, we measured and recorded temperature and relative humidity using strategically placed data loggers. These instruments continuously recorded microclimatic conditions at regular intervals throughout the study period, ensuring comprehensive coverage of temporal variability. In addition to temperature and humidity, we measured wind speed using the same data‐logging devices, which recorded all variables simultaneously. Including wind speed allowed us to better characterize local microclimatic conditions and assess their potential influence on bird presence and behavior (Rahman et al. 2025).

### 2.3 Vegetation Surveys

Concurrently with the bird surveys, we assessed vegetation structure using the quadrat method at multiple spatial scales: trees were surveyed within 10 × 10 m quadrats, shrubs within 5 × 5 m quadrats, and grasses and herbs within 1 × 1 m quadrats. In total, we sampled 80 plots, with 20 plots per park used to quantify herbaceous and shrub layers (Rahman et al. 2024, 2025). We identified and recorded all plant species within each quadrat, collecting detailed data on species richness, plant height, canopy cover, and ground cover.

These fine‐scale vegetation measurements are essential for linking plant community composition to bird habitat preferences and for interpreting patterns in bird community structure and behavior. By applying standardised and widely accepted field techniques, we ensured the consistency and reliability of vegetation and avian data across all study sites.

### 2.4 Statistical Methods

We used NMDS ordination and PERMANOVA analyses in R (version 4.4.2) using the vegan package (Oksanen et al. 2007) with Bray–Curtis dissimilarity. Community compositional dissimilarities among plots and parks were visualised using NMDS, while differences in community composition among parks were assessed using PERMANOVA. Means, standard deviations, and frequency tables were also used to summarize bird abundance data. Vegetation data described in Section 2.3 were used for contextual site characterization; however, the formal integration of vegetation variables as predictors will be addressed in a future study. All analyses were conducted using a significance threshold of *α* = 0.05 (R codes provided in the Data S1).

## 3 Results

### 3.1 Bird Species Richness

**FIGURE 2:**
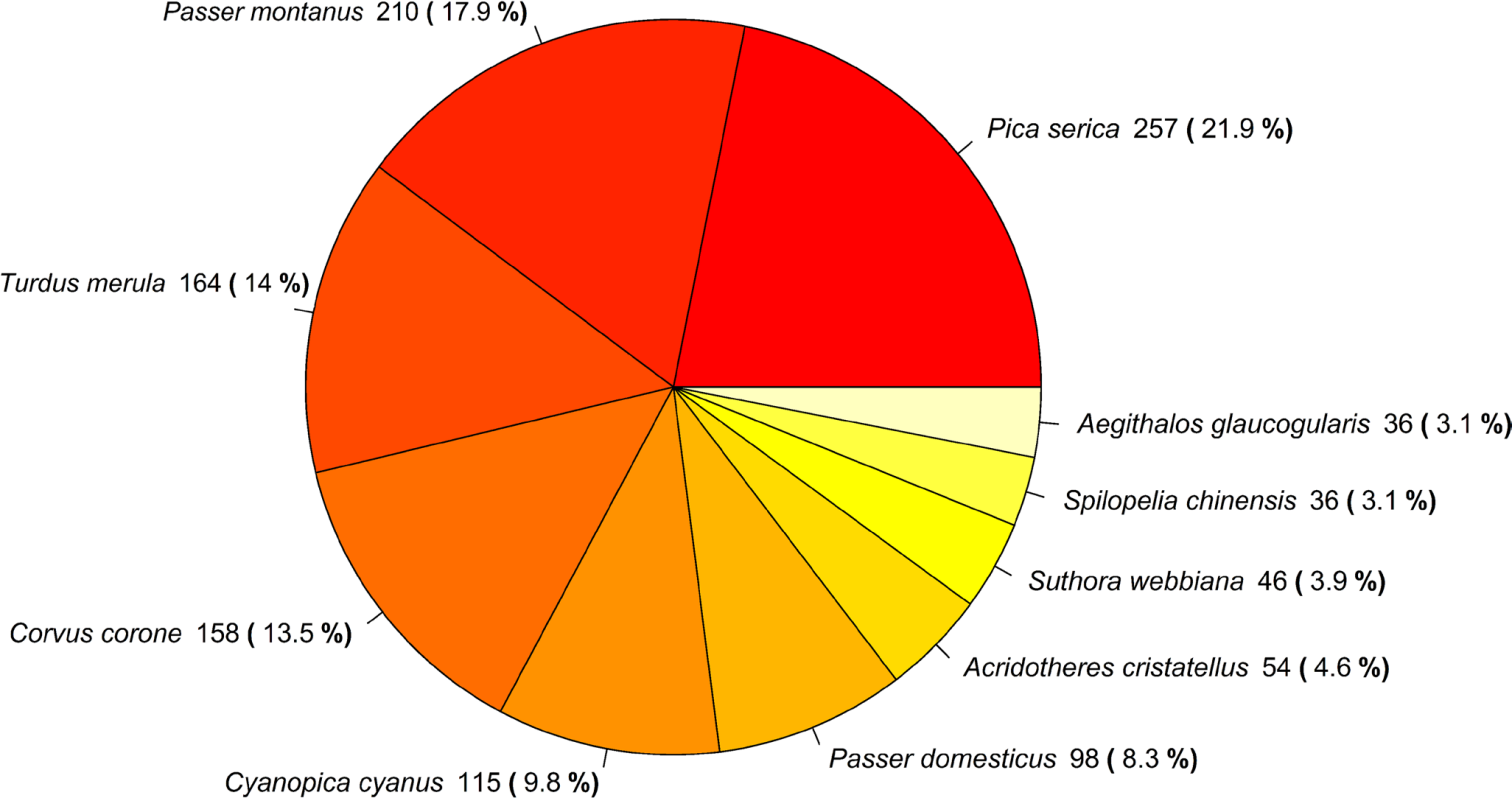
Relative abundance of the top 10 most frequently observed bird species in all the urban parks.

We found 68 bird species included in 25 families in 80 plots spread out in Beijing Olympic Park, Dongba Country Park, Chaoyang Park, and Wenyuhe River Park. The number of species in each park increased from 23 to 41, with Wenyuhe River Park exhibiting the highest number. Among the observed species in all the urban parks, we discovered that *Pica serica* (21.9%) was the most abundant, followed by *Passer montanus* (17.9%) and *Turdus merula* (14%). *Corvus corone* (13.5%) and *Cyanopica cyana* (9.8%) also represented substantial portions of the bird community, while *Acridotheres cristatellus* (4.6%), *Suthora webbiana* (3.9%), *Spilopelia chinensis* (3.1%), and *Aegithalos glaucogularis* (3.1%) accounted for smaller percentages (Figure 2).

The highest seasonal peaks in population were found for *P. montanus* , *P* *.* *serica* and *Columba livia* , all city‐dwelling and seed‐eating species. Instead, *Spatula clypeata* and *Acrocephalus tangorum* were found in just a few parks at specific times. We found *Nycticorax nycticorax* , *Egretta garzetta* and *Anser cygnoides* spotted in the Wenyuhe River Park. We detected several species listed on the national conservation list, including *A. cygnoides* (Endangered) and *A. tangorum* (Vulnerable). *P. montanus* , *P. serica*, *Dendrocopos major* and *T. merula* were found regularly in all seasons and parks. Alternatively, many birds, such as *Falco amurensis* , *Spatula clypeata* and *Cuculus canorus* were primarily found in spring and autumn. Some bird species, such as *A. tangorum* and *Ardea cinerea*, were only found in Wenyuhe River Park during spring and summer.

### 3.2 NMDS Analysis

**FIGURE 3:**
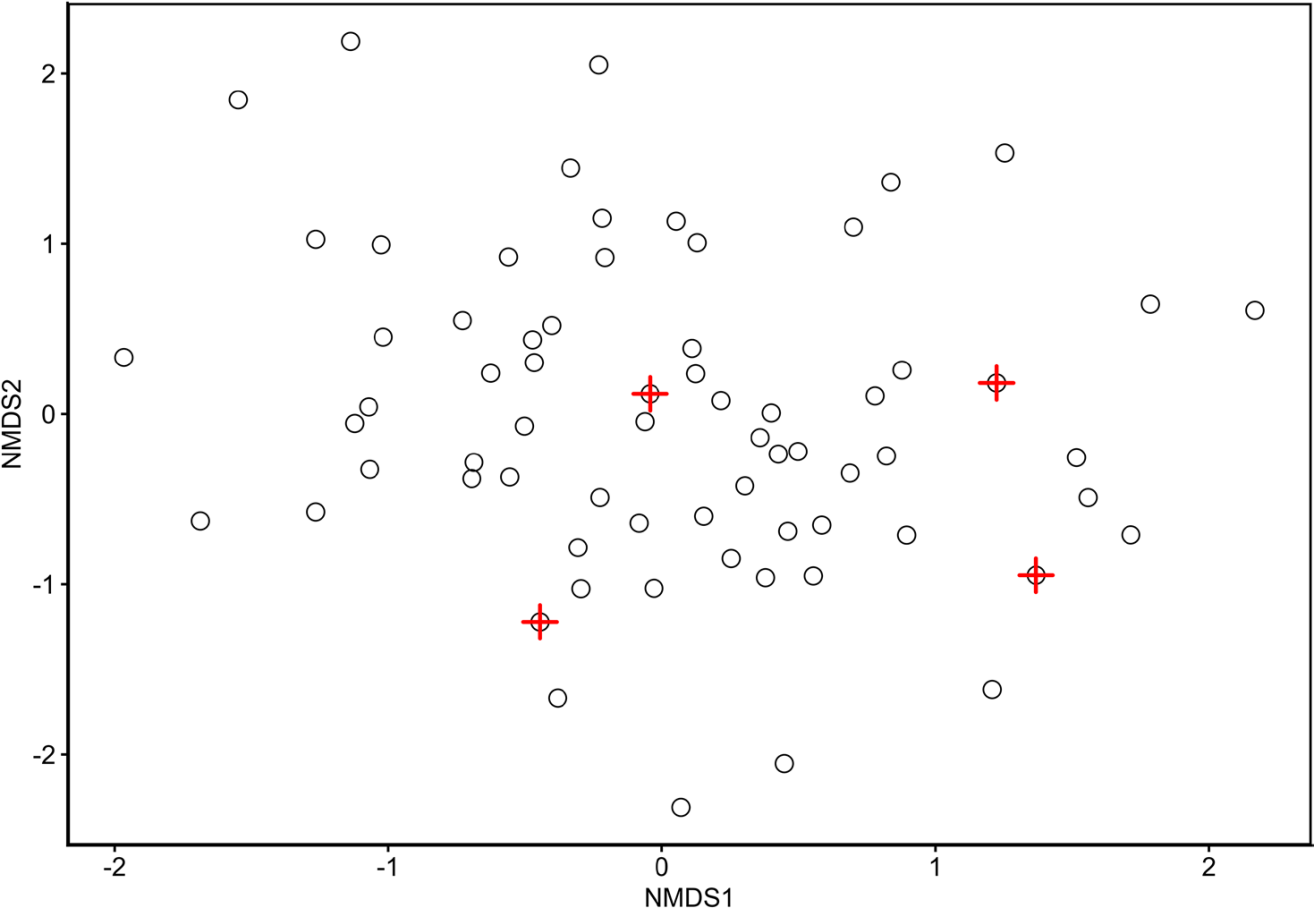
Nonmetric Multidimensional Scaling (NMDS) ordination plot.

To demonstrate how species in each sample were related and changed in avian community composition among the various habitats, we carried out an NMDS analysis. The ordination outcomes gave low levels of stress, which showed that the data were well represented in reduced dimensions. The stress values of NMDS1 and NMDS2 were 0.07 and 0.06, respectively, which indicated that ordinations were able to reflect the underlying ecological gradients in the models. In NMDS analysis, we found that most of the samples cluster close to the center, implying the presence of similar communities, but those with a wider variety of species are in the peripheral areas (Figure 3). The positions of the groups in the ordination are marked by the red plus signs and could be a reference to treatments or the center point of the treatment. The variability of the various components of the same environment is clearly rediscovered in our results, which show the ecological patterns and relationships among the sampled population.

**FIGURE 4:**
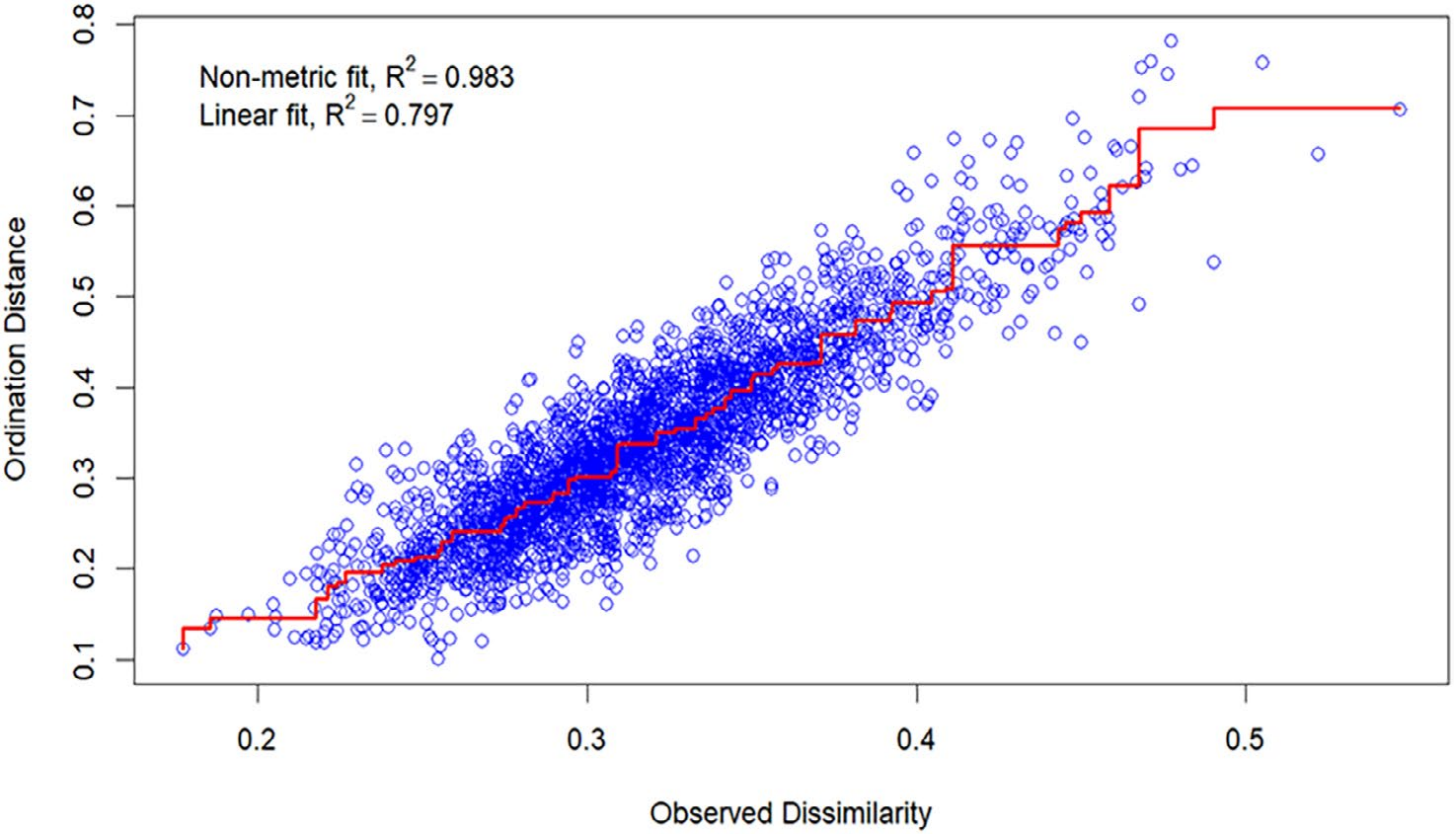
Stress plot (Shepard diagram) evaluating the Nonmetric Multidimensional Scaling (NMDS) ordination fit.

Furthermore, we showed the connection between the differences observed in the samples (*x*‐axis) and the ordination distances between those samples (*y*‐axis) (Figure 4). Every blue circle in the chart compares two samples to show their actual dissimilarity alongside their distance from each other in the new NMDS space. The red line represents a monotonic regression, which demonstrates that the ordination preserves the relative differences in the data. The high, nonmetric *R* ^2^ value indicates that the NMDS effectively preserves the differences in similarity among cases, rendering the two‐dimensional outcome a reliable representation of the actual multivariate pattern. NMDS includes rank order and does not place less emphasis on the linear *R* ^2^, but it still demonstrates a strong association. Overall, the results indicate that the ordering from the NMDS closely aligns with the dissimilarity data.

**FIGURE 5:**
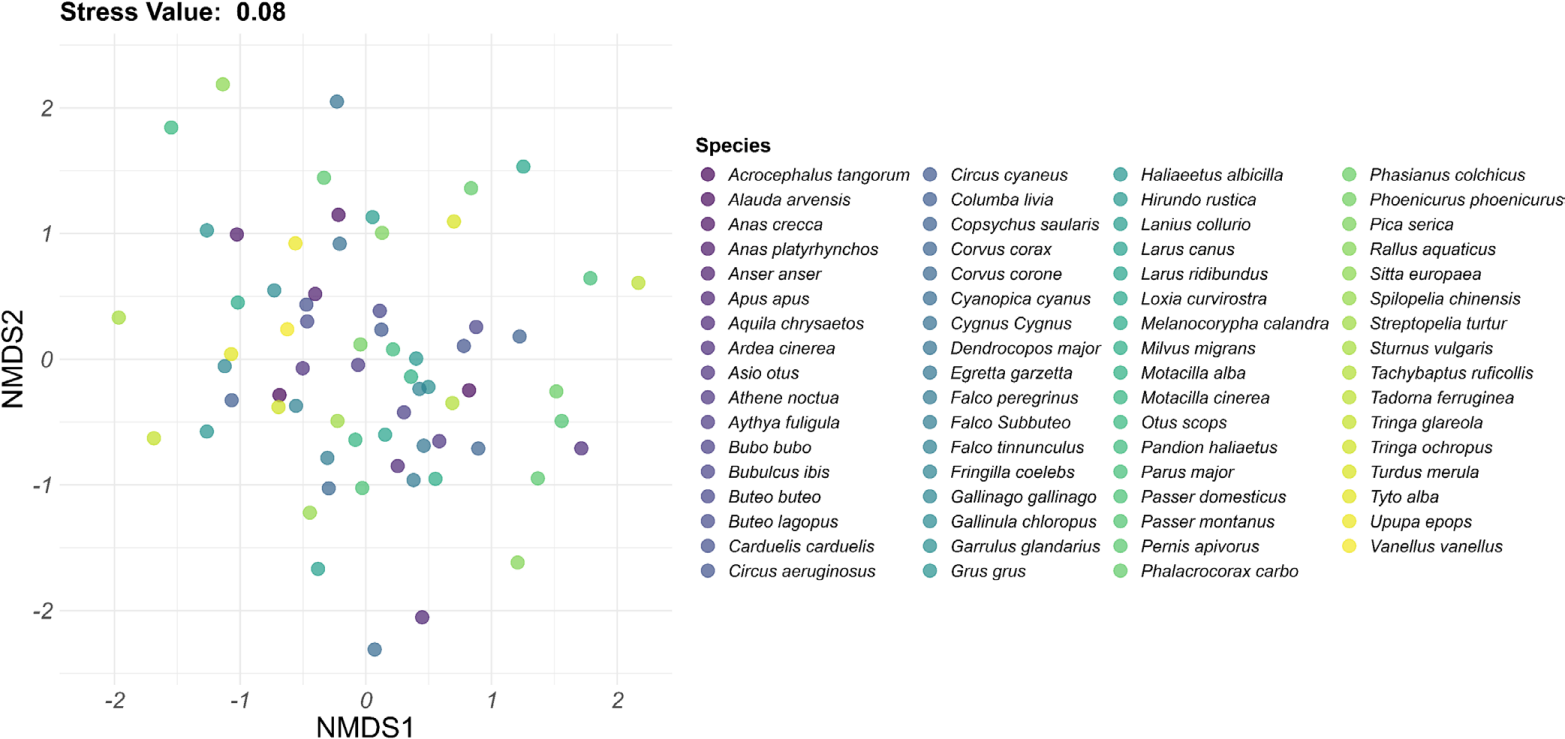
Nonmetric Multidimensional Scaling (NMDS) ordination plot color coded by the park. Our results of The PERMANOVA results also indicate that there are statistically different community compositions among the various groups. The proportion of variance due to park variance is just slightly more than 10% (*R* ^2^ = 10.78%) and the variance within parks accounts for 89.22% of the variance in the data (Table 1). We also remark that the *R* ^2^ is relatively small, at 10.78%, indicating that the entire effect of the park is not so much what distinguishes the park from other urban green spaces as it is a measure of the within‐park variation.

We used NMDS ordination plot designed to show how different species in each sample contribute to the overall ecological dissimilarity (Figure 5). Every dot is a sample, and the varying colors group them by habitat type, park, or season. The closeness of two samples in a two‐dimensional space reflects their shared number of species—samples that are near each other in the diagram have similar species communities. When many points are located near the center, we observe that the samples exhibit identical community structures, whereas those scattered far apart represent differences in community structure. The low value of stress in the top‐left corner (0.083) indicates that the ordination accurately reflects the differences without significant distortion. Using color‐coded points against a grid makes it very clear how the composition of each group stands out among the others.

An *F*‐value of 3.06 indicates some separation between the centres of each group, and the *p*‐value of 0.001 confirms that these differences are statistically significant (Table 1). Three of the 79 degrees of freedom are given to the grouping factor, and the residuals have 76. The total sum of squares is found to be 3.717, and the model explains 0.400701 of it. According to these results, the type of group (e.g., park, habitat, treatment) is a statistically significant influencing factor for community structure. The PERMANOVA results for each park pair show that the bird communities in urban parks are statistically different (Table 2).

**TABLE 1:** PERMANOVA results in comparing bird community composition among four urban parks.

df	SumOfSqs	*R* ^2^	*F*	Pr(>*F*)
3	0.400	0.107	3.060	0.001
76	3.317	0.892		
79	3.717	1		

Abbreviation: df, degree of freedom.

**TABLE 2:** Pairwise PERMANOVA comparisons between urban parks using Bray–Curti's dissimilarity.

Pairs	df	SumsOfSqs	*F*.Model	*R* ^2^	*p*	*p* adjusted
Beijing Olympic Park versus Chaoyang Park	1	0.171	3.815	0.091	0.001	0.006*
Beijing Olympic Park versus Wenyhue river park	1	0.148	3.095	0.075	0.001	0.006*
Dongba country park versus Chaoyang Park	1	0.126	3.210	0.077	0.001	0.006*
Dongba country park versus Wenyuhe river park	1	0.146	3.461	0.083	0.001	0.006*
Chaoyang Park versus Wenyhue river park	1	0.084	1.913	0.047	0.002	0.012

Abbreviation: df, degree of freedom.

* Statistically significant.

The table presents pairs of parks, and for each comparison, the *F*‐statistic, explained variance (*R* ^2^), the raw *p*‐value, and an adjusted *p*‐value (corrected using the Bonferroni method) are provided to aid in determining the significance of the changes (Table 2). Every comparison between pairs of parks reveals substantial differences, and their adjusted *p*‐values are all below 0.05. As an illustration, Beijing Olympic Park and Chao‐yang Park exhibit extreme compositional differences, as evidenced by the highest *F*‐value (3.82) and the greatest explained variation (*R* ^2^ = 0.091). The *F*‐value and *R* ^2^ measures for these parks are moderately to highly significant, indicating that their biological communities remain distinct. The least difference is shown between Chaoyang Park and Wenyuhe (Chaoyang Park–Wenyuhe, *R* ^2^ = 0.047, *F* = 1.91). Although significant (*p* = 0.002, adjusted *p* = 0.012), this suggests that these two areas are less ecologically distinct from others (Table 2).

## 4 Discussion

Our research provides useful information about the types of birds and their habitats in various urban green spaces throughout Beijing. We demonstrate, using NMDS and PERMANOVA, that changes in location and season have a significant influence on the diversity of bird species in city parks. Because of these findings, more people are beginning to recognize urban parks as areas that are important for both people and wildlife and capable of hosting diverse life forms (Aronson et al. 2014).

### 4.1 Ecological Differentiation Among Urban Parks

It appears from the NMDS and PERMANOVA that bird communities in each park are strongly influenced by the habitats present within those parks. There were *A. cygnoides* (endangered) and *A. tangorum* (vulnerable) species, such as in Wenyuhe River Park, with its wetland habitat, which attracted many wetland‐dependent and threatened species. Our result matches that of Fernández‐Juricic and Jokimäki (2001) and Shanahan et al. (2015), who found that urban wetlands differ significantly from others due to their environment. The PERMANOVA test (*R* ^2^ = 10.78%, *p* = 0.001) also shows consistent, statistically significant differences in composition among parks but does not provide information on the mechanisms driving this difference. The low *R* ^2^ also indicates that variation between parks accounts for a relatively small portion of the total variation in the community, and most of the variation is due to the within‐park variation. These variations could be due to the complexity of vegetation, the distribution of water, or the extent to which humans alter the environment—topics that have long been studied in urban bird ecology (Wang et al. 2013; Smeraldo et al. 2020). Bird communities are more diverse and interesting in parks with wetland borders and multilevel trees (Strohbach et al. 2009).

### 4.2 Dominance of Urban Generalists and Biotic Homogenization

All parks were found to be characterised by many urban‐living generalists, especially *P. montanus* , *P. pica* and *C. livia* . As a result of these traits—being flexible in their habitats, accepting humans nearby, and utilising urban food sources, these birds are successful in cities worldwide (Marzluff 2017; McKinney 2006). The consistent dominance of these generalists across all four parks is consistent with patterns of biotic homogenization, where a few adaptable species increase in relative abundance in cities, often at the expense of habitat specialists (Olden et al. 2004). However, we note that we did not formally calculate a β‐diversity index to confirm homogenization in this dataset; this remains a priority for future analysis. Functional diversity—the variety of ecological roles and trait combinations present in the community—can persist even where taxonomic diversity is constrained (Aronson et al. 2017). The scarcity of specialist species more likely reflects habitat unsuitability than active competitive exclusion by generalists, as most parks lack the undisturbed structural complexity required by many specialists.

### 4.3 Season for Migratory Connectivity

When *F. amurensis* and *C. canorus* come to urban parks, their seasonal presence is consistent with these parks serving as potential stopover habitats during migration; however, without direct evidence from capture‐recapture, tracking, or body‐condition studies, we cannot confirm active stopover use (La Sorte et al. 2018; Seewagen and Slayton 2008). Although people often view cities as environmentally harmful, we found that in the presence of water and native plants and insects, city parks can provide a refuge for migratory birds and their prey. According to recent evidence, urban green spaces, regardless of their level of fragmentation, may still serve as practical pathways for migratory animals to cross the city. Therefore, these results suggest that migratory birds need native plants and areas designed for each season in parks (González‐Oreja et al. 2020).

### 4.4 Conservation Opportunities in the Urban Matrix

Cities require more conservation planning, especially since Beijing's parks host threatened species. Typically, preserving green space volume has been the top concern in urban conservation; however, according to our research, the most crucial factor is the ecological quality, especially when wetlands, native plants, and features involving water are considered (Goddard et al. 2010; Aronson et al. 2014). Wenyuhe River Park's presence challenges the traditional distinction between urban areas and natural spaces, supporting the idea that cities can serve as a haven for nature. When biodiversity is considered in local planning, zoning, and infrastructure, it can enhance the city's resilience and improve the delivery of essential services (Francis et al. 2009). Management priorities for some of these parks include the establishment of natural greenways, the control of artificial light and noise levels, and the monitoring of invasive species, such as exotic turf grasses already recorded in these areas, including *Poa pratensis* . It should be emphasised that urban parks cannot be considered substitutes for natural habitats, as noise, artificial lighting, and human disturbance may negatively affect reproductive success and favor species that are more tolerant of anthropogenic stress (Francis et al. 2009). The absence of ground‐nesting species in our surveys may reflect their regional rarity, but it could also be partly explained by habitat unsuitability caused by disturbance from visitors, dogs, and routine park maintenance activities. Proactive management measures will therefore be essential to mitigate biodiversity loss as urban areas continue to expand.

### 4.5 Limitations and Future Directions

While the results of this study are well supported by the data, several limitations should be acknowledged. Hidden, nocturnal, and canopy‐dwelling species may have been underrepresented because of their low detectability during visual surveys. In addition, some passerine species may not have been adequately represented in the supplementary afternoon surveys (3:00–4:00 p.m.), suggesting that future surveys should focus primarily on early morning counts or incorporate acoustic recording devices. Another important limitation is the absence of nonurban reference sites, such as nature reserves or rural woodlands, which prevents direct comparisons between urban and nonurban environments and limits causal inference regarding the effects of urbanization. Furthermore, without capture–recapture methods, tracking data, or nest records, it was not possible to determine stopover use by migratory species or confirm breeding activity in threatened species such as *A. cygnoides* . Predator–prey dynamics, reproductive success, and the role of urban parks as population sources or sinks also remain important open research questions.

Finally, the inclusion of functional, phylogenetic, and taxonomic diversity metrics, as proposed by Flynn et al. (2009), could further strengthen future investigations. Further research should establish a system to regularly reassess both the total population and the number of places occupied. If we incorporate acoustic sensors, drone‐based habitat mapping, and environmental DNA (eDNA), it could sharpen our data. Moreover, by utilising platforms like eBird, the range of data can be expanded and shared among community members (Sullivan et al. 2014).

## 5 Conclusions

This study emphasizes the ecological importance of urban parks in Beijing and provides a comprehensive overview of the bird communities inhabiting these green spaces. Our results, which reveal substantial seasonal and spatial variation in bird diversity, suggest that urban parks not only serve as refuges for wildlife but also function as key corridors for migrating species. These findings highlight the critical need to preserve and enhance urban green spaces to support biodiversity in cities. Furthermore, our research opens avenues for future studies to explore the role of urban parks in fostering species interactions, migration patterns, and ecological resilience. The methods employed, such as NMDS and PERMANOVA, can be applied to other urban areas, contributing to the broader understanding of urban ecology. Future research should focus on integrating more advanced monitoring technologies, expanding biodiversity metrics, and establishing long‐term monitoring systems to better assess the impact of urbanization on wildlife. Ultimately, these results will help guide sustainable urban planning and contribute to the creation of green spaces that support both wildlife and human well‐being. Future research should address potential underrepresentation of nocturnal, hidden, and tree‐dwelling species by incorporating advanced monitoring techniques, such as acoustic sensors, drone‐based habitat mapping, and eDNA sampling. Expanding the study to include functional, phylogenetic, and taxonomic diversity would offer deeper insights into species' ecological roles. Additionally, establishing a long‐term monitoring system, with regular assessments of bird populations and habitat use, would help track changes over time. Collaborating with platforms like eBird could enhance data collection and foster community involvement. These efforts will strengthen urban biodiversity conservation and support sustainable green space management in cities.

## Author Contributions

**Anees Ur Rahman:** conceptualization (equal), data curation (equal), formal analysis (equal), investigation (equal), methodology (equal), writing – original draft (equal), writing – review and editing (equal). **Luciano Bosso:** supervision (lead), validation (lead), visualization (lead), writing – original draft (lead), writing – review and editing (lead). **Shahid Ahmad:** conceptualization (equal), data curation (equal), investigation (equal), methodology (equal), supervision (equal), visualization (equal), writing – original draft (equal), writing – review and editing (equal). **Najeeb Ullah:** investigation (equal), methodology (equal), visualization (equal), writing – original draft (equal), writing – review and editing (equal). **Muhammad Zahid:** conceptualization (equal), data curation (equal), funding acquisition (equal), investigation (equal), validation (equal), visualization (equal), writing – original draft (equal), writing – review and editing (equal). **Rashid Rasool Rabbani Ismaili:** investigation (equal), methodology (equal), visualization (equal), writing – original draft (equal), writing – review and editing (equal).

## Funding

This work was supported by National Recovery and Resilience Plan, CN_00000033.

## Conflicts of Interest

The authors declare no conflicts of interest.

## Supporting information


**Data S1:** NMDS R code.

## Data Availability

The data and codes are available in the main paper and in Data S1 or are freely accessible through the links referenced in the main text.
