# Supplementary material for: Urban Parks Act as Refuges for Avian Biodiversity in Chaoyang District, Beijing (China)
Source: Ecol Evol. 2026 Jul 2;16(7):e73953. doi: 10.1002/ece3.73953 (PMC13327796; doi:10.1002/ece3.73953)
Supplement: Supplementary file 1 — Data S1: NMDS R code. [file ECE3-16-e73953-s001.docx]

**Supplementary Material**

**NMDS R code**

library(sf)

library(dplyr)

library(ggplot2)

library(ggspatial)

# =============================================================================

# DATA PREPARATION

# =============================================================================

# Assume all sf objects are already loaded and projected consistently:

# gps_sf_proj, chaoyang_main, chaoyang_park_sf_proj, dongba_park_sf_proj, wenhue_park_sf

# Transform Wenhue River Park points to Chaoyang CRS

wenhue_park_sf_proj <- st_transform(wenhue_park_sf, st_crs(chaoyang_main))

# =============================================================================

# POINT CLASSIFICATION WITH PRIORITY

# =============================================================================

# Start with all GPS points

gps_points_unique <- gps_sf_proj %>% mutate(category = NA)

# Priority assignment (highest to lowest):

# 1. Chaoyang Park

in_chaoyang_park <- lengths(st_intersects(gps_points_unique, chaoyang_park_sf_proj)) > 0

gps_points_unique$category[in_chaoyang_park] <- "GPS in Chaoyang Park"

# 2. Dongba Park

in_dongba_park <- lengths(st_intersects(gps_points_unique, dongba_park_sf_proj)) > 0 & is.na(gps_points_unique$category)

gps_points_unique$category[in_dongba_park] <- "GPS in Dongba Park"

# 3. Wenhue River Park

in_wenhue_park <- lengths(st_intersects(gps_points_unique, wenhue_park_sf_proj)) > 0 & is.na(gps_points_unique$category)

gps_points_unique$category[in_wenhue_park] <- "GPS in Wenhue River Park"

# 4. Rest go to Chaoyang District

in_chaoyang_district <- lengths(st_intersects(gps_points_unique, chaoyang_main)) > 0 & is.na(gps_points_unique$category)

gps_points_unique$category[in_chaoyang_district] <- "GPS in Chaoyang District"

# Remove any points outside all polygons

gps_points_unique <- gps_points_unique %>% filter(!is.na(category))

# =============================================================================

# POLYGON LAYER PREPARATION

# =============================================================================

# Assign layer names to each park polygon

chaoyang_main$layer <- "Chaoyang District"

chaoyang_park_sf_proj$layer <- "Chaoyang Park"

dongba_park_sf_proj$layer <- "Dongba Park"

wenhue_park_sf_proj$layer <- "Wenhue River Park"

# Combine all park polygons for plotting

parks_poly <- rbind(

chaoyang_main[, "layer"],

chaoyang_park_sf_proj[, "layer"],

dongba_park_sf_proj[, "layer"],

wenhue_park_sf_proj[, "layer"]

)

# =============================================================================

# PLOTTING - CHAOYANG DISTRICT MAP

# =============================================================================

p <- ggplot() +

# Park polygons with transparency

geom_sf(data = parks_poly, aes(fill = layer), color = "black", alpha = 0.3) +

# GPS points with priority-based coloring

geom_sf(data = gps_points_unique, aes(color = category),

size = 2.8, shape = 21, stroke = 0.6, fill = "white") +

# Fill colors for polygons

scale_fill_manual(values = c(

"Chaoyang District" = "gray85",

"Chaoyang Park" = "deepskyblue3",

"Dongba Park" = "darkorange",

"Wenhue River Park" = "mediumorchid"

)) +

# Point colors (your requested color scheme)

scale_color_manual(values = c(

"GPS in Chaoyang District" = "#1f78b4", # Blue

"GPS in Chaoyang Park" = "#e31a1c", # Red

"GPS in Dongba Park" = "#33a02c", # Green

"GPS in Wenhue River Park" = "#ff7f00" # Orange

)) +

# Map extent

coord_sf(xlim = st_bbox(chaoyang_main)[c("xmin", "xmax")],

ylim = st_bbox(chaoyang_main)[c("ymin", "ymax")]) +

# Map decorations

annotation_scale(location = "bl", width_hint = 0.5) +

annotation_north_arrow(location = "bl", which_north = "true",

style = north_arrow_fancy_orienteering) +

# Labels

labs(title = "Chaoyang District Parks and GPS Points",

subtitle = "GPS points colored by park location with priority assignment",

fill = "Park Boundaries",

color = "GPS Points") +

# Theme

theme_minimal(base_size = 14) +

theme(legend.position = "right")

# Display the plot

print(p)

# =============================================================================

# SAVE HIGH-RESOLUTION MAP

# =============================================================================

ggsave("Chaoyang_Parks_GPS_Priority.tiff",

plot = p,

dpi = 600,

width = 10,

height = 8,

units = "in",

device = "tiff",

compression = "lzw")

# =============================================================================

# BIRD OBSERVATION BAR PLOT (Your previous code)

# =============================================================================

library(tidyverse)

# Prepare and clean bird observation data

df <- my_all_data_on_birds_in_one_sheet %>%

select(Species = `Scientific name...1`, Observations) %>%

mutate(Observations = as.numeric(Observations)) %>%

drop_na()

# Summarize and compute percentages

bird_summary <- df %>%

group_by(Species) %>%

summarise(Observations = sum(Observations)) %>%

mutate(Percentage = round(100 * Observations / sum(Observations), 2)) %>%

arrange(desc(Percentage))

# Create horizontal bar plot with frame

bar_plot <- ggplot(bird_summary, aes(x = Percentage, y = reorder(Species, Percentage))) +

geom_col(fill = "forestgreen") +

geom_text(aes(label = paste0(Percentage, "%")), hjust = -0.1, size = 2.5) +

labs(title = "Bird Species Observation Percentage",

x = "Percentage (%)", y = "Species") +

xlim(0, max(bird_summary$Percentage) + 5) +

theme_minimal() +

theme(

panel.border = element_rect(color = "black", fill = NA, size = 0.8),

plot.background = element_rect(color = "black", fill = NA, size = 1),

plot.margin = margin(10, 10, 10, 10)

)

# Display bar plot

print(bar_plot)

# Save bar plot

ggsave("bird_observation_bar_plot.tiff",

plot = bar_plot,

width = 8,

height = 6,

dpi = 600)

# =============================================================================

# DOT PLOT WITH FRAME (Your previous code)

# =============================================================================

dot_plot <- ggplot(bird_summary, aes(x = Percentage, y = reorder(Species, Percentage))) +

geom_point(color = "darkred", size = 3) +

geom_text(aes(label = paste0(Percentage, "%")), hjust = -0.3, size = 2.5) +

labs(title = "Bird Species Observation Percentages",

x = "Percentage (%)", y = "Species") +

theme_minimal() +

theme(

panel.border = element_rect(color = "black", fill = NA, size = 0.8),

plot.background = element_rect(color = "black", fill = NA, size = 1),

plot.margin = margin(10, 10, 10, 10)

)

# Display dot plot

print(dot_plot)

# Save dot plot

ggsave("bird_observation_dot_plot.tiff",

plot = dot_plot,

width = 8,

height = 6,

dpi = 600)

# =============================================================================

# PIE CHART - TOP 10 SPECIES (Your previous code)

# =============================================================================

top_10 <- bird_summary %>% slice_max(Percentage, n = 10)

pie_chart <- ggplot(top_10, aes(x = "", y = Percentage, fill = Species)) +

geom_col(width = 1, color = "white") +

coord_polar(theta = "y") +

geom_text(aes(label = paste0(Percentage, "%")),

position = position_stack(vjust = 0.5),

size = 3) +

labs(title = "Top 10 Bird Species - Pie Chart") +

theme_void() +

theme(

panel.border = element_rect(color = "black", fill = NA, size = 0.8),

plot.background = element_rect(color = "black", fill = NA, size = 1),

plot.margin = margin(10, 10, 10, 10),

legend.position = "right"

)

# Display pie chart

print(pie_chart)

# Save pie chart

ggsave("bird_observation_pie_chart.tiff",

plot = pie_chart,

width = 6,

height = 6,

dpi = 600)

# =============================================================================

# DISPLAY ALL PLOTS IN SEPARATE WINDOWS

# =============================================================================

# Map plot

dev.new(width = 10, height = 8)

print(p)

# Bar plot

dev.new(width = 8, height = 6)

print(bar_plot)

# Dot plot

dev.new(width = 8, height = 6)

print(dot_plot)

# Pie chart

dev.new(width = 6, height = 6)

print(pie_chart)
